# Treatment and Clinical Outcome of a Patient With Spindle Cell Rhabdomyosarcoma Harboring MEIS1-FOXO1 Gene Fusion

**DOI:** 10.7759/cureus.89124

**Published:** 2025-07-31

**Authors:** Rebecca Mathew, Rachel K Voss, Arash O Naghavi, Evita Henderson-Jackson, Andrew S Brohl

**Affiliations:** 1 Department of Chemistry, University of South Florida, Tampa, USA; 2 Department of Sarcoma, Moffitt Cancer Center, Tampa, USA; 3 Department of Radiation Oncology, Moffitt Cancer Center, Tampa, USA; 4 Department of Anatomic Pathology, Moffitt Cancer Center, Tampa, USA

**Keywords:** foxo1, fusion, meis1, molecular diagnostics, rhabdomyosarcoma

## Abstract

Fusion-driven extraosseous spindle cell rhabdomyosarcoma (SRMS) is a rare and recently recognized subcategory of rhabdomyosarcoma, with limited data on optimal management and clinical outcomes. We present the clinical course and long-term outcome of a unique case of SRMS harboring a novel *MEIS1-FOXO1* gene fusion diagnosed in a 40-year-old female. The case was successfully managed with a treatment regimen including surgery, radiation, and chemotherapy following a low-risk rhabdomyosarcoma paradigm. This report highlights the importance of molecular diagnostics in identifying rare gene fusions in SRMS and may help guide the management of future patients.

## Introduction

Rhabdomyosarcoma (RMS) is a subtype of sarcoma defined by its skeletal muscle lineage. Though rare, RMS is the most common soft tissue sarcoma to affect the pediatric population, with approximately 350 new cases diagnosed in the United States each year [1]. RMS is subdivided into four histologic subtypes, including embryonal and alveolar, the most common pediatric subtypes, pleomorphic, which is a rare sarcoma mostly seen in adults [2], and spindle cell rhabdomyosarcoma (SRMS). Spindle cell rhabdomyosarcoma (SRMS) is a rare and clinically diverse subgroup of this already uncommon soft tissue malignancy that is increasingly subdivided by molecular findings [3]. “Fusion-driven extraosseous SRMS” is one of the more recent of these disease sub-categorizations, owing to the increasing utilization of next-generation sequencing for molecular diagnostics and the resultant identification and reporting of rare driver fusion variants. Given this recency and rarity, treatment details and clinical outcomes are lacking. Therefore, it is incumbent to review and report this information to help guide the treatment of future patients. At present, there are approximately 30 cases of fusion-driven extraosseous SRMS reported in the literature, with a wide variety of driver fusions described [3]. Among reported cases is the case of a patient with a novel *MEIS1-FOXO1 *fusion, which, to our knowledge, is the only reported case of this fusion event in this disease [4]. Here, we provide an extended description of the clinical treatment and follow-up of this patient with a unique *MEIS1-FOXO1 *fusion SRMS. 

## Case presentation

**Figure 1:**
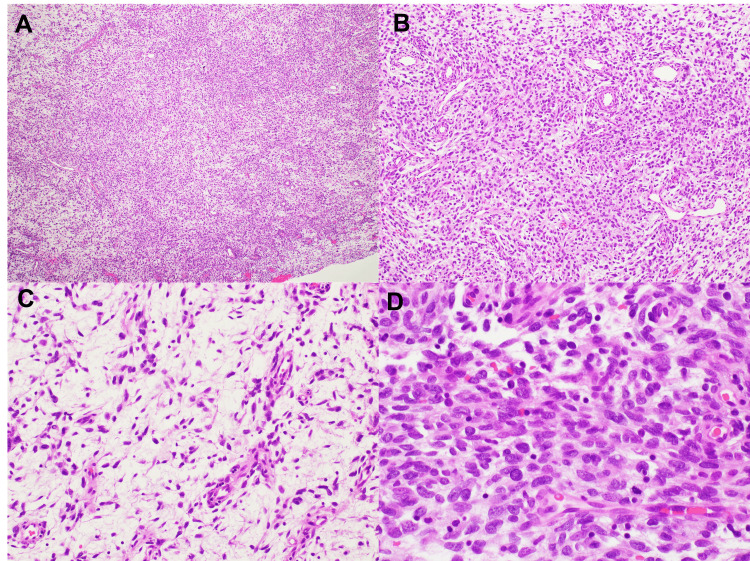
Tumor histology following hematoxylin and eosin staining. A) At low power microscopic examination (4x), the tumor demonstrates a mixed appearance of solid cellular areas and areas with stromal myxoid change.   B) At medium power (10x), solid cellular areas comprised of ovoid/spindle-shaped cells with indistinct, lightly eosinophilic cytoplasm are present, and rare mitoses are noted.  C) 20x magnification of a less cellular area demonstrates stromal myxoid changes with stellate/spindle cells with scant eosinophilic cytoplasm and mild to moderate nuclear pleomorphism. Background vasculature has the resemblance of arborizing vessels, characteristic of a myxofibrosarcoma-like histology.  D)  At high power (40x), increased nuclear: cytoplasm ratio and nuclear pleomorphism are apparent.

The patient is a 40-year-old female with a past medical history of tobacco abuse and uterine leiomyoma who presented with a left inguinal soft tissue mass initially misdiagnosed as an inguinal hernia. An open hernia repair was planned with no preoperative imaging. During this procedure, the surgeon identified a “cystic lesion with gelatinous material” within the inguinal canal, measuring 6 cm in aggregate, that was removed in piecemeal fashion and sent for pathologic evaluation. Pathological review of this specimen was consistent with an extraosseous SRMS with myxofibrosarcomatous features (Figure 1). A *MEIS1-FOXO1 *fusion (exon 6::exon 2) was identified by next-generation sequencing as previously described [4]. 

Following referral to our center, staging imaging with pelvic magnetic resonance imaging (MRI) and positron emission tomography/computed tomography (PET/CT) scans demonstrated no metastatic disease and no obvious residual disease in the surgical bed. A 523-gene targeted next-generation sequencing panel (TruSight Oncology 500, Illumina, Inc., San Diego, USA) was performed on the surgical specimen, which demonstrated no putatively pathogenic secondary mutations and a low mutational burden (0.0 mutations/Mb). The patient was treated with vincristine, dactinomycin, and cyclophosphamide (VAC/VA) x 8 cycles, as per the low-risk subset 1 group of ARST0331 [5]. Following the third chemotherapy cycle, the primary site was managed with neoadjuvant radiation (4500-5000 cGy in 25 fractions), covering the tumor bed with a 1.5 to 3cm margin along the inguinal canal, areas of potential surgical seeding during the hernia repair, along with any suspicious femoral lymph nodes.

She then underwent radical re-resection of the prior left inguinal scar, entire left inguinal canal (including the residual distal round ligament), a portion of the left rectus muscle, and the inguinal ligament, followed by immediate reconstruction with a biologic mesh and a left anterolateral thigh flap. No residual tumor was identified on pathology. Following surgical recovery, the planned systemic treatment course was completed and was well-tolerated.

Following the completion of therapy, the patient remains in active surveillance and is without evidence of disease recurrence with a follow-up duration of 39 months from the time of the initial diagnosis.

## Discussion

Spindle cell rhabdomyosarcoma (SRMS) is a rare disease subgroup that is increasingly molecularly defined. We report extended clinical treatment and outcomes details for a case of SRMS harboring *MEIS1-FOXO1 *gene fusion, to our knowledge, the only case reported in the literature. The clinical outcome for this patient was favorable following a low-risk RMS treatment paradigm.

The clinicopathological features of this case align with prior reports of SRMS and are perhaps most akin to other rare extraosseous SRMS tumors that harbor *MEIS1-NCOA2 *fusions, having a similar myxofibrosarcoma-like histology [4,6]. These tumors, though uncommon, are part of a growing body of evidence that highlights the molecular and clinical diversity of SRMS. The clinical behavior of extraosseous SRMS driven by rare fusions such as *MEIS1-NCOA2* or *MEIS1-FOXO1* is still being explored. Congruent with our reported case, early data suggest that fusion-driven extraosseous SRMS may have a more favorable prognosis compared to more common but still rare molecular subtypes of SRMS, such as MyoD1 mutated SRMS and fusion-driven intraosseous SRMS [3]. Given the limitations of isolated case studies, further reports are needed to verify our observations for *MEIS1-FOXO1* fusion RMS specifically. 

With the evolving nature of sarcoma classification, this case highlights the importance of next-generation sequencing to fully characterize rare driver gene fusion events in RMS. In this disease, *FOXO1 *rearrangements are typically associated with alveolar rhabdomyosarcoma (ARMS), an aggressive and poor prognostic subtype, in which *FOXO1 *is paired with either *PAX3 *or *PAX7 *[7]. The clinicopathological features of the *MEIS1-FOXO1* SRMS case described clearly differ from the typical case of *PAX3/7-FOXO1* fusion ARMS. It is likely that SRMS, particularly the extraosseous fusion-driven molecular subtype, carries a more favorable prognosis. Therefore, for RMS cases with atypical histology or clinical presentation, such as older age or spindle cell histology, testing for *FOXO1 *gene rearrangements should include characterization of the fusion gene partner to avoid mischaracterization of all “*FOXO1-*rearranged” tumors as ARMS.

## Conclusions

We report an extended clinical report of a unique case of spindle cell rhabdomyosarcoma that harbors *MEIS1-FOXO1* fusion. With more than 3 years of follow-up, the clinical outcome of this case remains favorable following treatment using a low-risk RMS paradigm. The clinicopathological features of this case are consistent with prior reports of SRMS and are perhaps most akin to other rare *MEIS1-NCOA2* extraosseous cases. Though rare, this case highlights that testing for *FOXO1 *gene rearrangement ideally should include characterization of the fusion gene partner to avoid mischaracterization of all “*FOXO1-*rearranged” tumors as alveolar RMS. Ideally, collaborative research will help to further clarify the clinical implications of rare fusion events such as *MEIS1-FOXO1* in this disease.
